# The Pacific Tree-Parasitic Fungus *Cyclocybe parasitica* Exhibits Monokaryotic Fruiting, Showing Phenotypes Known from Bracket Fungi and from *Cyclocybe aegerita*

**DOI:** 10.3390/jof7050394

**Published:** 2021-05-19

**Authors:** Hannah Elders, Florian Hennicke

**Affiliations:** Project Group Genetics and Genomics of Fungi, Chair Evolution of Plants and Fungi, Ruhr-University Bochum, 44780 Bochum, Germany; hannah.elders@ruhr-uni-bochum.de

**Keywords:** Basidiomycota, multicellular development, morphogenesis, basidiocarp, fructification, reproduction mode, plant-pathogenic fungi, life cycle

## Abstract

*Cyclocybe parasitica* is a wood-destroying parasitic edible mushroom growing on diverse broad-leafed trees in New Zealand and other Pacific areas. Recent molecular systematics of European *Cyclocybe aegerita*, a newly delimited Asian phylum and of related species, corroborated the distinction of the chiefly saprobic cultivated edible mushroom *C. aegerita* from *C. parasitica*. Here, we show that *C. parasitica* exhibits a morpho-physiological trait characteristic to its European cousin, i.e., monokaryotic fruiting *sensu stricto* (basidiome formation without mating). Monokaryotic fruiting structures formed by *C. parasitica* ICMP 11668-derived monokaryons were categorized into four phenotypes. One of them displays ulcer-like structures previously reported from bracket fungi. Histology of dikaryotic and monokaryotic *C. parasitica* fruiting structures revealed anatomical commonalities and differences between them, and towards monokaryotic fruiting structures of *C. aegerita*. Mating experiments with *C. parasitica* strains representative of each fruiting phenotype identified compatible sibling monokaryons. Given reports on hypothetically monokaryotic basidiome field populations of ‘*C. aegerita sensu lato*’, it seems worthwhile to prospectively investigate whether monokaryotic fruiting s.str. occurs in nature. Sampling from such populations including karyotyping, comparative -omics, and competition assays may help to answer this question and provide evidence whether this trait may confer competitive advantages to a species capable of it.

## 1. Introduction

*Cyclocybe parasitica* (G. Stev.) Vizzini (syn. *Agrocybe parasitica* G. Stev.) is a wood-destroying edible tree-parasitic Pacific agaric that has been recorded to grow on living individuals of diverse broad-leafed trees. Its occurrence has been recorded in various Pacific areas including New Zealand and Australia [1,2,3,4], and reportedly also in Hawaii, Mexico, Colombia, and China [5]. Watling [6] stated that *C. parasitica* is widespread and certainly replaces its mainly saprobic European relative(s) *Cyclocybe aegerita* (V. Brig.) Vizzini/*Cyclocybe cylindracea* (DC.) Vizzini & Angelini [7] in New Zealand, and possibly also in parts of Australia [8]. To date, the species status of *C. aegerita* (syn. *Agrocybe aegerita* (V. Brig.) Singer) towards *C. cylindracea* (syn. *Agrocybe cylindracea* (DC.) Maire) is still under debate for a resilient clarification of which a rigid reevaluation has been recommended recently by Frings et al. [7]. This unclear status, however, caused floristic studies recording the presence of this originally European species in Europe and on other continents such as Asia and South America to apply different species names. While Singer [9] favored the species epithet ‘*aegerita*’, Watling & Gregory [10] and Nauta [11] disagreed using an incorrect [12] epithet based on the older name. Since both names, i.e., *C. aegerita* and *C. cylindracea*, are currently valid [7], here, for the time being, we render all records outside Europe, also the ones by authors applying the epithet based on the older name, as putative records of ‘*C. aegerita sensu lato*’.

*Cyclocybe aegerita* from Europe is a white-rot fungus primarily degrading dead wood of deciduous trees [11,13,14]. It is also a cultivated edible mushroom in many countries that fruits in consecutive flushes [7,15]. The latter pattern is reflected in the expression of fruiting-related genes (FRGs) as recently shown by Orban et al. [16]. Moreover, *C. aegerita* serves as a model species to study crucial basidiomycete mushroom life cycle-related topics such as mating and fruiting [13,17,18]. Recent molecular systematics combined with differing morpho-physiological features yielded a clear delimitation of European *C. aegerita* from the Pacific species *C. parasitica* and other relatives. By this, Frings et al. [7] confirmed classic field-mycological [1,11] and distribution-wise [8] differentiation of *C. aegerita/*’*C. aegerita* s.l.’ from *C. parasitica*. Basidiomes of *C. aegerita* from nature reach a cap size of 15 cm and a stipe length of 11 cm [11], while *C. parasitica* can reach respective sizes of 20–25 cm and of up to 20 cm [1,8]. On relative scale, these differences also seem to reproduce in axenic culture [7].

When originally describing *C. parasitica* from New Zealand, Stevenson [1] reported that it has most commonly been observed to grow on living individuals of the endemic Malvaceae tree taxa *Plagianthus betulinus* A. Cunn. and species from the genus *Hoheria* A. Cunn. Notably, it has also been recorded to infest a wide variety of other native and introduced broad-leafed trees, a couple of which are relevant as commercial timber trees in New Zealand, e.g., *Beilschmiedia tawa* Benth. & Hook. f., species of the genus *Populus* L., or *Platanus orientalis* L. [1,2,5,8]. *Beilschmiedia tawa* and *Dysoxylum spectabilis* Hook. f. in particular seem to be other common hosts of *C. parasitica*, which is reported as common and widespread in New Zealand [8]. Its pathogenic lifestyle is exemplarily underlined by the fact that the fungus can exploit its living host as lignocellulosic substrate over several decades, being associated with a very slow heart-rot. This way of host exploitation is reported to lead to a gradual dieback of the infected tree [1,8]. In addition, Stevenson [1] states that the fungus may not persist for long on dead wood of its host(s). Still, it is not clear how long the species may live on in dead substrate since Watling & Taylor [8] also reported it from dead wood. During the exploitation time of living hosts, the fungus regularly forms basidiomes that grow out either from high on the tree or from mechanical injuries such as clefts or insect-made pittings, e.g., larvae tunnels and flight holes of *Aenetus virescens* Doubleday, commonly known as puriri moth [1].

Watling and Taylor [8] consider the fungus to be almost certainly indigenous, and they record predominantly four-spored basidia with a rare observation of two-spored ones. The same applies to *C. aegerita* with respect to field mycological description of collected basidiomes from nature [11]. In contrast, Watling [6] mentions one southern Brazilian and three Argentinean collections of ‘*C. aegerita* s.l.’ with prominent two-spored basidia. In addition, he refers to a two-spored form of ‘*C. aegerita* s.l.’ Singer [19] described from western parts of Argentina and Chile. These observations seem very interesting in relation to the fact that laboratory-reared true homokaryotic fruiter (THF)-type fruiting bodies of *C. aegerita* feature two-spored basidia. Their basidiospores are postulated to be a product of mitosis, with no mating and meiosis involved [17,18,20]. In contrast to this case of monokaryotic fruiting *sensu stricto* [21], (homo)dikaryotic basidiomes of *C. aegerita* display four-spored basidia composed of dikaryotic hyphae [11,18], the same as homodikaryotic ones formed by pseudohomokaryotic fruiters (PHF) [17]. According to the field observations of Nauta [11], Singer [19], Watling and Taylor [8], and Watling [6], a potential occurrence of monokaryotic fruiting s.str. by ‘*C. aegerita* s.l.’ in nature alongside regular dikaryotic fruiting cannot be excluded *a priori*.

Thus far, the capability of monokaryotic fruiting and the anatomy of respective fruiting structures has only been studied in *C. aegerita* (only counting in strains of certified European origin or identity as *C. aegerita*). Thus, capability of monokaryotic fruiting in ‘*C. aegerita* s.l.’ from outside Europe and related species remains to be investigated. Hence, in order to acquire experimental data about the distribution of this morpho-physiological trait among the relatives of *C. aegerita*, we set out to test the Pacific species *C. parasitica* for its capability to form monokaryotic fruiting structures.

## 2. Materials and Methods 

### 2.1. Strains, Culture Maintenance, and Nuclear State Verification

Twelve progeny monokaryons of dikaryon *C. parasitica* ICMP 11668 [7], i.e., *C. parasitica* ICMP 11668-1 to -12 were obtained via single basidiospore isolation and germination in the same way as described previously. Moreover, routine propagation, long-term storage, and confirmation of the nuclear state (to ascertain that they are truly monokaryotic) of *C. parasitica* ICMP 11668-1 to -12 was carried out as described for *C. aegerita* [18], with minor modifications. Micro-cultivation chamber-grown micro-cultures of *C. parasitica* ICMP 11668-1 to -12 on glass slides were first fixated with fixation solution (3:1 ethanol/acetic acid) for 10 min. Afterwards, the slides were washed with water several times, until they were stained with Calcofluor and 4′,6-diamidino-2-phenylindole (DAPI) as described by Herzog et al. [18]. For fruiting experiments, dikaryon *C. aegerita* AAE-3 and fruiter type monokaryon *C. aegerita* AAE-3-32 [18] were used as positive controls. 

### 2.2. Fruiting Setup for C. parasitica ICMP 11668 and Its Progeny Monokaryons

For fruiting, four replicate 1.5% MEA plates per strain were centrally inoculated with a mycelium overgrown agar plug (0.3 cm diameter, punched out from the edge of a freshly colonized 1.5% MEA plate of the respective strain). Of each quadruplicate of plates per strain, one plate was used to monitor the growth speed of each strain. The other three replicates were wrapped in aluminum foil and grown until the control replicate had reached the plate edge. Plates inoculated with *C. parasitica* were pre-incubated at 25 °C in the dark for 13 days (dikaryon *C. parasitica* ICMP 11668) or 15 to 19 days (monokaryons *C. parasitica* ICMP 11668-1 to -12), until mycelia reached the edge of the plates. Analogously, plates of the control strains *C. aegerita* AAE-3 and *C. aegerita* AAE-3-32 [18] were incubated at 25 °C in the dark for 10 and 14 days, respectively. Subsequently, the fully-grown plates were transferred to the fruiting conditions specified by Herzog et al. [18], with a few modifications: fruiting wet chambers were assembled using 70% ethanol-disinfected microboxes (Eco2 microboxes TP2100+TPD2100 from Eco2 NV, Geraardsbergen, Belgium). Saturated humidity within the boxes was achieved by placing two fresh paper towels on the bottom of each chamber and soaking it with 10 mL of sterile distilled water. These wet chambers were then each loaded with a triplicate of fully-grown 1.5% MEA plates per individual strain. Before that, an agar plug of 0.3 cm diameter was punched out and removed from each plate as an additional fruiting stimulus, about as performed previously [7,18]. Fruiting chamber assembly was completed by removing the Petri dish lids and placing them beneath the plates in two out of three replicate plates. To have all three replicate plates placed on a platform within the fruiting chamber, we placed the lid of a sterile 60 × 15 mm Petri dish beneath the third culture plate. The prepared fruiting chambers were incubated in a 12 h white light/12 h darkness regime at 20 °C using a growth chamber (PK-520 growth chamber, polyklima GmbH, Freising, Germany) equipped with True Daylight PLUS LEDs (polyklima GmbH). Light intensity was measured with a light meter (LI-250A from LI-COR Biosciences GmbH). A medium light intensity of 10 ± 0.5 μmol m^−^^2^ s^−^^1^ was detected. Fruiting experiments were repeated at least two times independently for each strain while each round comprised three replicates per strain.

### 2.3. Cross Sections

Microtome sections of fruiting structures formed by *C. parasitica* ICMP 11668 and its monokaryotic progeny monokaryons were prepared as previously described for *C. aegerita* [18], with a few minor modifications: cross sections of 8 µm thickness were prepared using a Rotary Supercut RM 2065 Microtome (Leica Mikrosysteme Vertrieb GmbH, Wetzlar, Germany) with a cutting speed of 50%. Sections were attached to glass slides with n-propanol [22]. The prepared slides were placed on a 50 °C heating block until they tightly stuck to the glass, and they were then stained with toluidine blue O as described by Herzog et al. [18]. Stained sections were dried again and covered with M-GLAS (Merck KGaA, Darmstadt, Germany) and a cover slip. Microscopic examination and composite image assembly was carried out at 200× magnification using an Axio Imager M2 (Carl Zeiss GmbH, Jena, Germany) equipped with a filter set 02 (Carl Zeiss GmbH, item number 488002-9901-000, suitable, e.g., for nuclear state fluorescence microscopy as applied in the present manuscript) and ZEN Works software version 2.6. 

### 2.4. Mating of Selected C. parasitica Monokaryons

Mating compatibility among the fruiting-wise most interesting *C. parasitica* ICMP 11668 progeny monokaryons was assessed using micro-cultivation chambers (MCs) that were assembled as described by Herzog et al. [18]. After an incubation time of 4 days at 25 °C, the overlap area of the two grown mycelia was examined microscopically (Axio Imager. M2 from Carl Zeiss Microscopy Deutschland GmbH) at 1000× magnification. The presence of clamp connections on hyphae indicated dikaryotization, meaning that the respective monokaryons should carry different allelic mating-type specificities.

## 3. Results

### 3.1. Fruiting Phenotypes of C. parasitica ICMP 11668 and Its Monokaryotic Progeny

**Figure 1:**
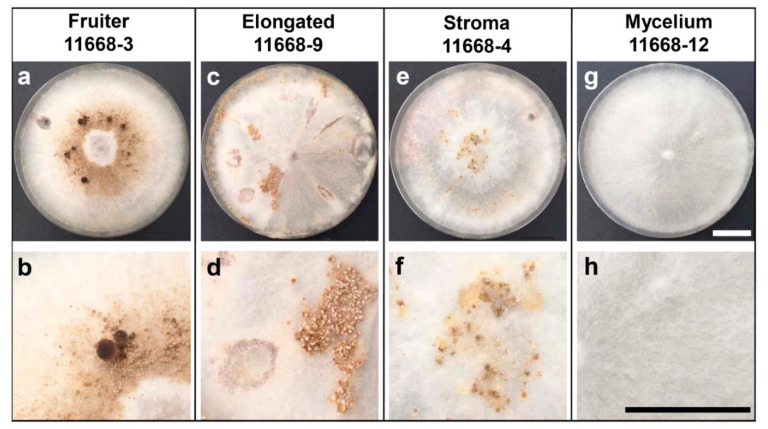
Spectrum of fruiting phenotypes by monokaryotic progeny of *C. parasitica* ICMP 11668. Strains were reared in the fruiting setup by Herzog et al. [18]: they were grown on 1.5% MEA for 16–19 days at 25 °C in the dark. Fully colonized plates were fruiting-induced at 20 °C in a 12 h light/12 h dark rhythm at saturated humidity in microfilter-aerated wet chambers. The different fruiting phenotypes are displayed by respective monokaryons exhibiting them. The fruiter type strain *C. parasitica* ICMP 11668-3 displayed monokaryotic basidiomes (**a**,**b**), strain *C. parasitica* ICMP 11668-9 exhibited elongated structures (**c**,**d**), and strain *C. parasitica* ICMP 11668-4 formed bloated structures (**e**,**f**) that Esser et al. [24], in a different species, referred to as ‘stromatic structures’ or ‘stromata’. All other strains did not form any fruiting structures, only displaying monokaryotic mycelium. They are represented by *C. parasitica* ICMP 11668-12 (**g**,**h**). The scale bar represents 2 cm.

First of all, the monokaryotic state of all here-employed *C. parasitica* ICMP 11668-derived monokaryons was confirmed by DAPI/Calcofluor staining (Figure S1). Remarkably, in contrast to *C. aegerita* AAE-3 [18] where spores germinate after 1–2 days, monokaryotic hyphae grew out from germinating spores only 14 days post-inoculation (p.i.). The dikaryon *C. parasitica* ICMP 11668 [7] and its monokaryotic progeny was then submitted to the fruiting regime of Herzog et al. [18], with the above-described modifications. As a control, the European dikaryon *C. aegerita* AAE-3 and its monokaryotic derivative *C. aegerita* AAE-3-32 were likewise submitted to this slightly modified fruiting regime. Fully developed fruiting structures were documented 21 days p.i. in *C. aegerita* AAE-3 (Figure S2a), 28 days p.i. in *C. aegerita* AAE-3-32 (Figure S2b), and 34 days p.i. in dikaryon *C. parasitica* ICMP 11668 (Figure S2c). These results align well with previous works [7,18]. Among all *C. parasitica* ICMP 11668-derived monokaryotic strains, fruiting bodies with a distinct cap and stipe were only formed by *C. parasitica* ICMP 11668-3, earliest 38 days p.i. (21 days post fruiting induction, f.i.). They developed in a pigmented brownish ring-like area of the mycelium around the inoculation plug and lacked full cap expansion. In consequence, the annulus that is characteristic for mature dikaryotic mushrooms by *C. parasitica* ICMP 11668 (see Figure S2c and [7]) will not become apparent with *C. parasitica* ICMP 11668-3 since the cap margin stays attached to the stipe (Figure 1a,b). Strain *C. parasitica* ICMP 11668-9 exhibited structures that are comparable to the morphologically similar phenotype from *C. aegerita*, i.e., the ‘stipe type’ of Meinhardt [23] and the ‘elongated initials type’ by Herzog et al. [18]. Those structures were documented earliest 43 days p.i. (27 days post f.i.). They formed in patches on the aerial mycelium of *C. parasitica* ICMP 11668-9, halfway to the edge of the plate. Just as observed by Herzog et al. [18], in *C. aegerita*, these elongated structures turn white towards their tip while they are brownish at the base. Another obvious feature of *C. parasitica* ICMP 11668-9 is that this strain exhibited a kind of radiating growth pattern by the aerial mycelium covering the plate (Figure 1c,d). Most remarkably, in *C. parasitica* ICMP 11668-4, another interesting fruiting type was observed, a type that has so far never been observed in the well-studied sister species *C. aegerita*. Nevertheless, this kind of monokaryotic fruiting structures has been observed previously in the bracket fungi *Polyporus ciliatus* Fr. and *Schizophyllum commune* Fr., being referred to as ‘stromata’, ‘stromatic structures’, or ‘stromatic proliferations’ [20,24]. In *C. parasitica* ICMP 11668-4, such structures were documented 76 days p.i. (57 days post f.i.). They are characterized by hard brownish ulcer-like structures forming in irregular patterns randomly distributed over the surface of the aerial mycelium in the respective culture plates. Moreover, in areas where many of these structures accumulated, exudations of a resin-like appearance were observed (Figure 1e,f). All other *C. parasitica* ICMP 11668-derived monokaryotic mycelia did not display any fruiting structures, which is representatively shown with *C. parasitica* ICMP 11668-12 (Figure 1g,h; Figure S2d–l).

### 3.2. Mating Compatibility of Fruiting-Competent Monokaryons

Mating compatibility assessment aimed at identifying at least one compatible mating partner for each of the fruiting phenotype-wise most interesting monokaryons *C. parasitica* ICMP 11668-3, -4, and -9. For this, these monokaryons were mated among each other and with all other here-employed sibling monokaryons. Mating in the closely related tetrapolar European species *C. aegerita* is thought to be controlled by a tetrapolar mechanism of homogenic incompatibility. In the model mushroom *S. commune*, the well-studied tetrapolar mating system is controlled by two unlinked loci *A* and *B* [25], as in *C. aegerita* [7,18,26]. Contact of mycelia of incompatible monokaryons sharing the same allelic specificities of the loci *A* and *B* results in mycelial growth indistinguishable from the one by the monokarytic mycelia [25]. Compatible mating reactions of partners carrying different allelic specificities of the mating type loci *A* and *B* (*A*≠, *B*≠) result in an establishment and outgrowth of dikaryotic hyphae with typical clamp connections [18,26]. These two features were, thus, considered a valid criterion for detecting mating compatibility of two *C. parasitica* monokaryons. Compatibility became apparent from the growth of distinct dikaryotic mycelium showing clamp connections after the colonies of the mating partners had grown into contact with each other. Mating of *C. parasitica* ICMP 11668-3 (fruiter type) revealed mating compatibility with two mycelium type monokaryons, i.e., *C. parasitica* ICMP 11668-6 and -8 as well as with the *C. parasitica* ICMP 11668-9 (elongated type). In the case of the stroma type monokaryon *C. parasitica* ICMP 11668-4, only one compatible mating partner was identified among *C. parasitica* ICMP 11668-1 to -12, i.e., *C. parasitica* ICMP 11668-5 (Figure S3). 

### 3.3. Fruiting Structure Anatomy in C. parasitica ICMP 11668 and Its Progeny

**Figure 2:**
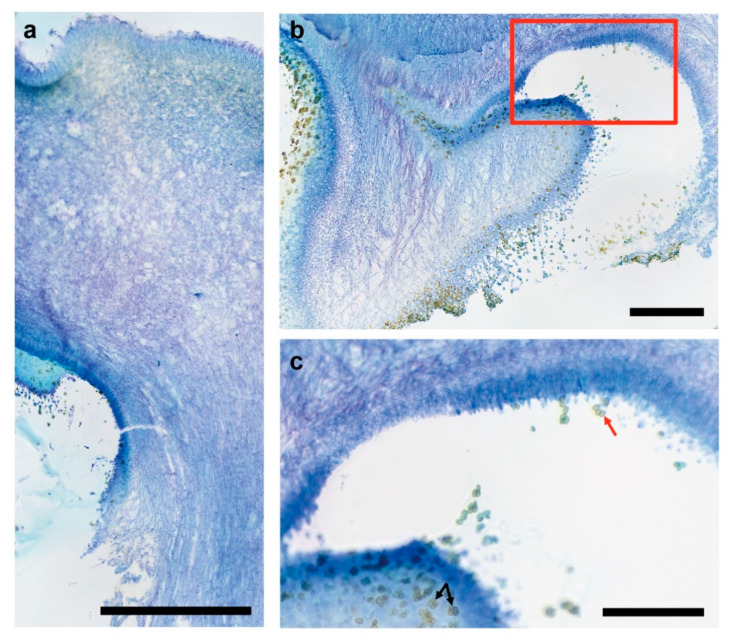
Anatomy of mature dikaryotic *C. parasitica* basidiomes. Cultures of *C. parasitica* ICMP 11668 were grown at 25 °C in the dark for 13 days. Fruiting induction (f.i.) took place with fully colonized plates at 20 °C in a 12 h light/12 h dark setup at saturated humidity in microfilter-aerated wet chambers. Photographs were taken from toluidine blue O-stained microtome sections of mature dikaryotic fruiting bodies harvested 21 days after f.i. (**a**) Longitudinal section showing the tissue architecture including the cap surface (upper left), cap context, transition to the stipe tissue, and the gill cavity (lower left, lined by the dark blue stained hymenium releasing multitudes of basidiospores) of a mature fruiting body of *C. parasitica* ICMP 11668. The cap surface tissue displayed a palisade-like layer of hyphal ends, many of which tower above neighboring ones. The rupture of tissue on the lower edge of the picture was caused during sample processing. The scale bar represents 0.5 mm. (**b**) Transition zone between cap, gills, and stipe showing a not completely unfolded gill that is closest to the stipe. The red frame represents the area that is shown in Figure 2c on a larger scale. The scale bar represents 200 µm. (**c**) The hymenium, i.e., the layer of dark blue stained palisade-like arranged hyphal ends, releases myriads of basidiospores and exhibits typical spore tetrads (black arrows) on four-spored basidia (red arrow). The scale bar represents 100 µm.

Anatomy of fruiting structures by *C. parasitica* ICMP 11668, and by *C. parasitica* ICMP 11668-3, *C. parasitica* ICMP 11668-4, and *C. parasitica* ICMP 11668-9 was assessed by microscopy of cross sections. Sampling only included fully developed fruiting structures exhibiting their final morphology. In agreement with the work of Frings et al. [7], mature dikaryotic fruiting bodies of *C. parasitica* ICMP 11668 displayed a completely elongated stipe, a fully expanded cap, and mostly unfolded gills (see Figure S2c). Histology shows that the cap surface is made up by a palisade-like layer of hyphal ends (Figure 2a). In mature *C. parasitica* ICMP 11668 basidiomes, the universal veil has vanished while it still covers the complete surface of immature basidiomes, the same as in immature basidiomes of European *C. aegerita* AAE-3 (see [18]). Yet, the cap surface layer of mature *C. parasitica* ICMP 11668 basidiomes contains many hyphal elements that tower above adjacent ones (see Figure 2a). Macroscopically, this feature makes the brown cap surface appear velutinous. Moreover, some hyphae in the palisade show inflated terminal or penultimate segments. In addition, histology revealed that the gills in closest proximity of the stipe, as in *C. aegerita* (see [18]), are not fully unfolded but are covered with fertile hymenium, also releasing multitudes of basidiospores (Figure 2b). The latter also lines the surface of the transition zone between cap and stipe where it also contains four-spored basidia and displays conspicuous basidiospore tetrads (Figure 2c). 

**Figure 3:**
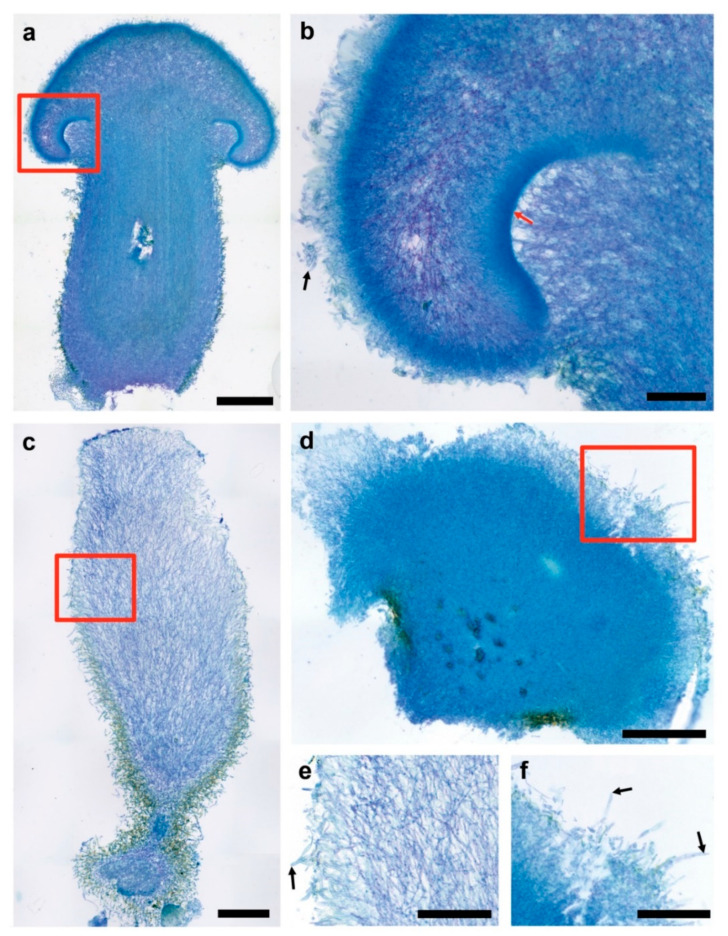
Anatomy of fully grown monokaryotic fruiting structures by *C. parasitica*. Cultures of *C. parasitica* ICMP 11668-3, -4, and -9 were grown at 25 °C in the dark for 16–19 days. Fruiting induction (f.i.) took place with fully colonized plates at 20 °C in a 12 h daylight/12 h dark setup at saturated humidity in microfilter-aerated wet chambers. Fully developed fruiting structures were harvested 21 days (*C. parasitica* ICMP 11668-3), 27 days (*C. parasitica* ICMP 11668-9), and 57 days (*C. parasitica* ICMP 11668-4) after f.i. Photographs were taken from toluidine blue O-stained microtome sections of fully developed fruiting structures. (**a**) Fully grown basidiome of *C. parasitica* ICMP 11668-3 (fruiter type). The tissue rupture in the center of the stipe was caused during the sample processing. The red frame represents the area that is shown in (**b**) on a larger scale. The scale bar represents 0.5 mm. (**b**) Image detail from (**a**) showing the gill cavity that is filled out by the partial veil but also displays a flat intensely stained hymenium (indicated by a red arrow). The black arrow points to hyphal elements of the prominent universal veil on the cap surface. The scale bar represents 100 µm. (**c**) Fully grown elongated fruiting structure formed by *C. parasitica* 11668-9 (elongated type) that exhibits an apportionment into a basal stand-like intensely stained condensed structure with a sheath-like pigmented layer lining its outer surfaces and a less dense less intensely stained upper part. The red frame represents the area that is shown in (**e**) on a larger scale. (**d**) Fully grown bloated fruiting structure formed by *C. parasitica* 11668-4 (stroma type) that shows a faint differentiation into a dense intensely blue strained internal tissue and a less dense outer tissue. The red frame represents the area that is shown in (**f**) on a larger scale. The scale bar represents 200 µm. The scale bar represents 200 µm. (**e**) Image detail from (**c**) showing the outer surface of an elongated structure by *C. parasitica* 11668-9 (elongated type) that contains partially inflated hyphal elements (black arrow). The scale bar represents 100 µm. (**f**) Image detail from (**d**) showing the outer surface of a bloated structure formed by *C. parasitica* 11668-4 (stroma type) that consists of partially inflated rather lilac-stained hyphal elements (black arrows). The scale bar represents 100 µm.

The *C. parasitica* ICMP 11668-derived monokaryons exhibited a wide spectrum of fruiting phenotypes. *Cyclocybe parasitica* ICMP 11668-3 exhibited fruiting bodies that resembled young dikaryotic basidiomes (Figure 3a). Still, cap-opening and gill development seem strongly reduced in the former ones. Moreover, these structures did not undergo a complete stipe elongation. The universal veil did not get thinned out on the outer surfaces of the fruiting bodies as much as it is typical for maturing dikaryotic hemiangiocarpic basidiomes, e.g., also in the related species *C. aegerita* [18]. Strikingly, the gill cavity—where the intensely stained hymenium stayed flat in profile (no gill development)—remained mostly filled out by the partial veil. Such is typical for early immature fruiting bodies as it has been illustrated with the European cousin of *C. parasitica*, i.e., *C. aegerita* [7,18]. In addition, fruiting bodies of *C. parasitica* ICMP 11668-3 did not produce any basidiospores (Figure 3b). On closer inspection, their stipe can be divided into three zones. A very thin dark bluish layer with an uneven surface (due to largely protruding hyphal ends, some of which seem partially embedded in blotches of a brownish pigment) makes up the outmost layer on the stipe surface. In some parts of this layer, yellowish-brownish stained patches can be noticed, especially towards the base of the stipe. Beyond the surface, a lilac intermediate zone is sharply delimited from an intensely blue inner zone. The delimitation becomes less sharp towards the top where the universal veil is separated from the partial veil (the future annulus) in the gill cavity by protruding inner tissue from the stipe (see Figure 3a). This three-zone segmentation was less apparent in the dikaryon due to the thinning out of the veil on the outer surfaces of the maturating basidiomes, as recorded for *C. aegerita* by Herzog et al. [18]. 

Apart from *C. parasitica* ICMP 11668-3, which produces sporeless abortive monokaryotic basidiomes, only two other *C. parasitica* ICMP 11668 progeny monokaryons formed fruiting structures. The elongated plectenchymatic structures formed by *C. parasitica* ICMP 11668-9 (Figure 3c) did not display any differentiation into distinct plectenchyma (‘tissue’) parts relating to a primordial cap or stipe. In comparison to the very compact internal tissue of the bloated structures by *C. parasitica* ICMP 11668-4 (Figure 3d) and of the abortive monokaryotic fruiting bodies by *C. parasitica* ICMP 11668-3 (see Figure 3a), the entire tissue of the elongated structures by *C. parasitica* ICMP 11668-9 appeared much less densely packed except for its most basal parts. Another conspicuous feature of this basal partition was its more or less stand-like appearance with a broad bed-plate and a constricted shaft-like structure that then transgresses into the club-shaped upper proportion of the elongated plectenchymatic structure. The elongated type also exhibits a zonation of reduced extent compared to the fruiter type. Here, a conspicuous outer zone, which forms a sheath-like intensely brownish-yellowish pigmented layer, can be distinguished. It extends halfway up to the apex of the elongated structure (see Figure 3c). The brown staining of the lower half of the elongated fruiting structures (in contrast to the white stained apical part) and velutinous appearance seems to reappear in the microscopic image (see Figure 3c). The downwards increasingly uneven outer surface of the fruiting structure consists a layer of partially swollen hyphae (Figure 3e). 

The bloated largely undifferentiated structures that are formed by *C. parasitica* ICMP 11668-4 (see Figure 3d) exhibited a densely packed and intensely bluish-stained tissue, which only loosens up in the outmost periphery. The intense blue color of the tissue gets paler and partially more lilac towards the surface layer. Similar to *C. parasitica* ICMP 11668-3 and -9, some yellowish-brownish stained patches of pigment were found to be present on parts of the fruiting structure tissue, especially at its surfaces. The upper outer layer of the displayed bloated fruiting structure of *C. parasitica* ICMP 11668-4 had a mostly uneven surface that consisted of rather lilac stained more or less protruding hyphal ends (Figure 3f). Some of them were terminally or subterminally inflated and more lilac-stained, consequently being of larger diameter than the hyphae in the inner tissue (see Figure 3d,f).

**Figure 4:**
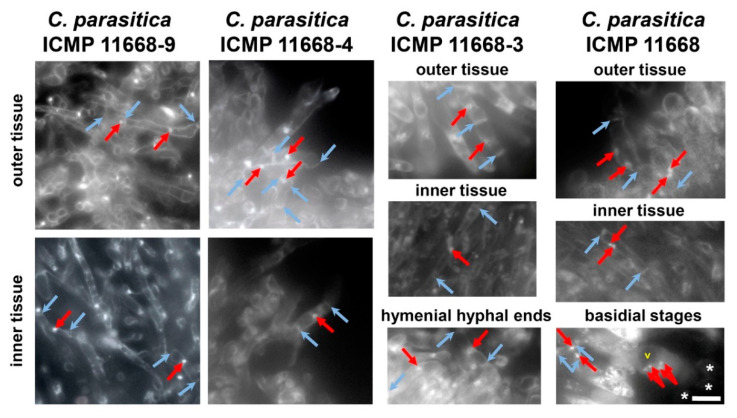
Nuclear state and septa allocation in fully grown dikaryotic and monokaryotic fruiting structures by *C. parasitica* ICMP 11668 progeny monokaryons. Cultures of *C. parasitica* ICMP 11668, 11668-3, -4, and -9 were grown at 25 °C in the dark for 13 or 16–19 days. Fruiting induction (f.i.) took place with fully colonized plates at 20 °C in a 12 h daylight/12 h dark setup at saturated humidity in microfilter-aerated wet chambers. Fully developed fruiting bodies with a distinct cap and stipe were harvested 21 days (*C. parasitica* ICMP 11668 and 11668-3), 27 days (*C. parasitica* ICMP 11668-9), and 57 days (*C. parasitica* ICMP 11668-4) after f.i. Photographs were taken from DAPI/Calcofluor-stained microtome sections of fully developed fruiting structures. Red arrows point to nuclei, barbed blue arrows indicate two septa delimiting a hyphal segment, white asterisks indicate basidiospores, a yellow ‘v’ marks a basidial vacuole. In the dikaryon *C. parasitica* ICMP 11668, outer tissue corresponds to the palisade-like layer of hyphal ends at the cap surface and inner tissue corresponds to cap tissue, both displaying dikaryotic hyphal segments without clamps. The picture depicting basidial stages of *C. parasitica* ICMP 11668 shows an already developed basidium in the hymenium that is connected to the hypha from which it arose by a simple septum (no clamp connection at the septum). In the fruiter type monokaryon *C. parasitica* ICMP 11668-3, outer tissue corresponds to hyphal ends at the lower cap margin surface while inner tissue corresponds to stipe tissue close to the tissue rupture (see Figure 3a). Both tissue types display monokaryotic hyphal segments without clamps. The picture depicting hymenial hyphal ends of *C. parasitica* ICMP 11668-3 shows that the hymenium is composed of simple-septated monokaryotic hyphal ends/segments. In the stroma type monokaryon *C. parasitica* ICMP 11668-4, outer tissue corresponds to the surface layer of hyphal ends on the upside of the fruiting structure. Inner tissue corresponds to tissue in the upper third of the intensely stained inner tissue. Both tissue types display monokaryotic hyphal segments without clamps. In the elongated type monokaryon *C. parasitica* ICMP 11668-9, outer tissue corresponds to the surface layer of hyphal ends on the right side in the transition area to the bed-plate of the fruiting structure. Inner tissue refers to tissue in the upper left third of the fruiting structure. Both tissue types show monokaryotic hyphal segments without clamps. The scale bar represents 10 µm.

In order to reconfirm that all fruiting structures formed by *C. parasitica* ICMP 11668-3, -4, and -9 are composed of monokaryotic hyphae, we applied nuclear and cell wall staining to selected unstained microtome sections of their fruiting structures. Via fluorescence microscopy, we found that both inner and outer plectenchyma of their fruiting structures is indeed composed of clampless monokaryotic hyphae compared to the situation in the dikaryon where these tissues of the basidiomes show clampless dikaryotic hyphae. In addition, it could be resolved that the basidia in the hymenium of dikaryotic fruiting bodies of *C. parasitica* ICMP 11668 arise from clampless dikaryotic hyphae, and that the hymenium of the monokaryotic basidiomes by *C. parasitica* ICMP 11668-3 is composed of simple-septated monokaryotic hyphal elements (Figure 4).

## 4. Discussion

### 4.1. Dikaryotic and Monokaryotic Basidiome Morphogenesis in C. parasitica 

Here, we have shown that the Pacific parasitic species *Cyclocybe parasitica* is able to undergo monokaryotic fruiting s.str., a fruiting mode that is thus far only known for its European mostly saprobic relative *C. aegerita*. In addition, we histologically resolved the anatomy of dikaryotic basidiomes of *C. parasitica* ICMP 11668 and of fruiting structures formed by three of its monokaryotic descendants. This revealed a fruiting phenotype that was previously known from the bracket fungi *S. commune* and *P. ciliatus*. Nuclear state examination of the hyphae composing the fruiting structures formed in the here-identified fruiting competent monokaryons *C. parasitica* ICMP 11668-3, -4, and -9 showed that these structures are composed of clampless monokaryotic hyphae. In contrast, the basidiome tissue of the dikaryon from which they originate is composed of clampless dikaryotic hyphae (see Figure 4), which is all congruent with what has been published for the related species *C. aegerita* [18]. Furthermore, this approach resolved the basidia in dikaryotic *C. parasitica* ICMP 11668 basidiomes to arise from clampless dikaryotic hyphae. Interestingly, if not eventually remaining sterile, it seems that another potential future *C. parasitica* basidium grows from the hyphal segment that is subapical to the already grown basidiospore-bearing basidium (see Figure 4). This is similar as in *S. commune*, where new basidia do not only arise from apical cells of hyphae but can also form from growing (subapical) clamp connections [27]. The formation of sporeless abortive monokaryotic basidiomes by *C. parasitica* ICMP 11668-3 (see Figure 1a and Figure 3a,b) proves that this species is, like *C. aegerita* [17,18], capable of abortive homokaryotic fruiting (AHF). A couple of major histological differences can be noted towards the AHF fruiting bodies of *C. aegerita* AAE-3-32. Such structures by *C. aegerita* AAE-3-32 exhibit a disproportionally flat cap and a reduced gill development. In contrast, the AHF basidiomes of *C. parasitica* ICMP 11668-3 display a total failure of gill formation and a more gibbous cap, with the cap margin still bent inwards towards the stipe. Both species’ AHF type basidiomes share an impairment of cap opening and a failure of partial veil separation from the cap margin (see Figure 1a and Figure 3a,b; Ref. [18]). On the basis of previous observations [17,18] in *C. aegerita*, we cannot exclude the possibility that *C. parasitica* ICMP 11668-3 may also be capable of forming monokaryotic basidiomes of the THF type (true homokaryotic fruiting). Moreover, since a certain strain will not always achieve fruiting in every fruiting flush on every single plate, it seems likely that, e.g., the stromatic proliferations by *C. parasitica* ICMP 11668-4, might form even earlier in future fruiting trials. Ultimately, Orban et al. [28] showed that some *C. aegerita* ‘mycelium-type’ monokaryons may prove themselves as (partially) fruiting-competent in setups other than the one by Herzog et al. [18]. Accordingly, this might also apply to *C. parasitica* ICMP 11668-derived monokaryons that did not form any monokaryotic fruiting structures here (see Figure S2d–l). Another notable characteristic of the monokaryotic fruiting by *C. parasitica* ICMP 11668-3 is the fruiting pattern this strain exhibits: its basidomes appear in a brownish ring-like area of the mycelium around the inoculation plug (see Figure 1a,b). To some extent, this fruiting pattern reminds us of the one by ring-type monokaryotic fruiters of *S. commune* [29], the genotypes of which can confer light-independent fruiting in a respective dikaryon. 

In comparison to the elongated initials formed by some *C. aegerita* AAE-3-derived monokaryons [18,28], elongated structures that are formed by *C. parasitica* ICMP 11668-9 seem to constitute something different. Although these structures look very similar to each other on the macroscopic level, both displaying a brown base and a white tip, microscopy reveals some noteworthy anatomical differences. In addition, potentially reflecting the radiating mycelium growth and the fruiting pattern by *C. parasitica* ICMP 11668 in the setup by Frings et al. [7], elongated structures of *C. parasitica* ICMP 11668-9 formed chiefly in the peripheral zone of the agar plates (see Figure 1c). In contrast, *C. aegerita* AAE-3-derived monokaryons such as *C. aegerita* AAE-3-22, representing the elongated initials type, seem to form their fruiting structures strongly concentrated at the plate edge and around the point of injury [18]. Moreover, the elongated structures of *C. parasitica* ICMP 11668-9 (see Figure 3c,e) consist of much less densely packed tissue than the elongated initials of, e.g., *C. aegerita* AAE-3-22 [18]. This much less densely packed tissue reminds us somewhat of a phenotype that has been observed in the bracket fungus *S. commune*. There, knockout mutants with a constitutive post-translational activation of Ras1 also exhibit a much less densely packed fruiting body tissue than the wild type [30]. It is possible that this congruence might relate to a misregulation of Ras1 or some kind of Ras1-dependent signaling within the genetic network, triggering monokaryotic fruiting in *C. parasitica* ICMP 11668-9. Another major difference between the elongated initials of *C. aegerita* AAE-3 and the elongated structures in *C. parasitica* is the stand-like shape with a basal bed plate that the latter ones exhibit (see Figure 3c). Potentially, this may just relate to phenotypic variability of some extent that such structures can exhibit, which may also be found in *C. aegerita*. Alternatively, this variability may relate to some deviant morphogenetic signaling exhibited by fruiting-related genes (FRGs) of *C. parasitica* over the one by the FRGs of *C. aegerita*, some of which have been annotated in the *C. aegerita* genome [16,31]. This could be revealed by comparative transcriptomic studies on these specimens in the future. The same scenario may apply to the differences in AHF-type fruiting structures of *C. aegerita* versus the ones of *C. parasitica*, compared to each other and in comparison to their dikaryotic counterparts. Such a cross-species global FRG expression landscape comparison might even benefit (e.g., allowing for an identification of potentially unknown FRG variants enabling fruiting in darkness) from including suitable *S. commune* strains exhibiting somewhat similar fruiting patterns such as the ring-type monokaryotic fruiters of Yli-Mattila et al. [29]. 

Ultimately, such comparative transcriptomics approaches might also help resolving the identity of the bloated structures formed by *C. parasitica* ICMP 11668-4 (see Figure 3d,f) towards the monokaryotic basidiome initials developed by *C. aegerita* AAE-3-derived monokaryons [18]. This way, one might obtain molecular evidence on the level of FRG expression at least with *C. parasitica* in how far one should consider such ‘stromatic proliferations’ as a dead end of multicellular development as proclaimed previously [20,24]. For both monokaryotic fruiting phenotypes, the ‘stroma type’ one in *C. parasitica* and the ‘elongated’ ones by *C. aegerita* and *C. parasitica*, a hypothetical lack of Dst1 [31,32,33,34] activity might lead to a block in cap tissue differentiation [16]. Further tissue proliferation, although in a polar way in the elongated types versus a non-directional one in stroma type structures, may then lead to the further increase in size of both types of structures over basidiome initials. 

### 4.2. Open Questions: Nature of Monokaryotic Fruiting s.str. and Its Potential Occurence in Wild Populations

Within the genus *Cyclocybe* Velen., monokaryotic fruiting s.str. has so far only been recorded for *C. aegerita* [17,18,21,28]. Its occurrence in *C. parasitica*, the parasitic Pacific cousin of the former species [7], shows that this feature is not restricted to a single species of the genus. On the broader perspective, the abundance of this peculiar morpho-physiological trait among the Agaricomycetes [20,35] still remains to be clarified, since monokaryotic fruiting s.str. excludes all cases which involve an at least not disproven homodikaryotization [21]. Moreover, recent studies touching upon the topic such as the one by Chen et al. [36] with *Volvariella volvacea* (Bull. ex Fr.) Sing., never histologically examined in how far they dealt with monokaryotic fruiting s.str. or s.l., or even a mixture of cases such as in the work of Labarère and Noël [17]. In the broader sense, this term involves homodi- and homomultikaryotization, the resulting hyphae of which are clampless in *V. volvacea*, while exhibiting clamps in *C. aegerita* [17,36]. Thus, homokaryotic basidiomes of fruiting-competent homokaryotic single spore isolates (SSIs) of *V. volvacea* by Chen et al. [36] can be assigned to the pseudohomokaryotic fruiter (PHF) type, a term Labarère and Noël [17] coined to describe homodikaryotic basidiomes formed by some *C. aegerita* strains. Amphithallic species such as *V. volvacea* [36] or *C. aegerita* employ a mixture of different mating mechanisms and life cycles. In *V. volvacea*, heterothallism (mating of self-sterile non-isogenic homokaryons germinated from mononucleate meiotic basidiospores) prevails, but secondary homothallism (as in *Agaricus bisporus* (Lange) Imbach) from binucleate meiotic heterokaryotic basidiospores is also evident. Similar to *C. aegerita* PHF strains in artificial culture [17], *V. volvacea* is capable of a homothallic life cycle by homomultikaryotization of fruiting-competent homokaryons growing from mononucleate meiotic homokaryotic basidiospores [36]. As in *C. aegerita* where PHF basidiomes exhibit four-spored basidia [17], basidia from basidiomes formed by *V. volvacea* hetero- or homokaryons are reported to be mostly four-spored (some are one-, two-, three-, and five-spored), showing typical meiotic stages and chiefly (>85%) mononucleate spores [37,38,39,40,41,42]. In contrast, monokaryotic fruiting s.str. is characterized by the formation of no or two-spored basidia with basidiospores that should originate from mitosis [17,18,20,21]. As a reproduction cycle, if not to be revealed as a form of unisexual or parasexual reproduction that generates genetic diversity via mitotic recombination, e.g., after endoreplication [43,44], monokaryotic fruiting s.str. seems completely asexual (no mating, no homodi-/multikaryotization). Thus, an intriguing question that remains is whether monokaryotic fruiters s.str. can actually establish populations of their own in nature? In the case of homokaryotic fruiters, this was to some extent discussed by Chen et al. [36] when arguing about the abundance of hetero- (outbreeding) versus homothallic (inbreeding) *V. volvacea* strains in nature. In the case of unisexual reproduction, it has even been shown that such a way of bypassing the sexual cycle can be useful. It does not only offer selective advantages (e.g., habitat exploration benefits), but even long-term drawbacks of such a bypassing of the sexual cycle can be reversed [45,46]. For the moment, the conundrum, whether agaric monokaryotic fruiting s.str., might dissemble some clever genetic trick(s) allowing a non-deleterious long-term bypassing of meiotic reproduction that seems so typical to mushrooms, cannot be solved on the basis of experimental data. 

Still, in the case of ‘*C. aegerita* s.l.’, Watling and Taylor [8] discuss that a presence of monokaryotic fruiters in nature may at least be one way of potentially resolving the presence of exclusively two-spored populations directly next to exclusively four-spored ones in South America. Such populations were encountered repeatedly, not only by Singer [19], but also by Watling and Taylor [8] and Watling [6]. By pointing to the works of Esser and co-workers [13,47,48], Watling and Taylor [8] suggest that pure cultures from such populations should be obtained to study these differences on the cellular level. 

Future works might benefit from applying a mixture of approaches. On the one hand, sampling, cultivation, and application of material from these South American locations to laboratory fruiting setups combined with histology and volatile profiling (e.g., the ones by Herzog et al. [18] and Orban et al. [28]) may be rewarding. On the other hand, colonization and fruiting rates on suitable wood-based substrates by dikaryons and already studied versus freshly isolated monokaryotic fruiters of *C. aegerita* and *C. parasitica* of different geographic origin may be assessed. This may allow some prediction on the reproductive success of hypothetical clonally reproducing populations of these species versus classically reproducing ones in nature. The experimental setup by Almasi et al. [49] using poplar logs might serve as a template for this.

## 5. Conclusions

In conclusion, it cannot be excluded that agaric monokaryotic fruiting s.str. might also take place in nature, hypothetically leading to an establishment of individual populations with two-spored basidia, as can be inferred from diverse field-based works. The fact that this trait is not only limited to ‘*Cyclocybe aegerita* s.l.’ but also exists in the widely spread Pacific parasitic species *C. parasitica* further corroborates this presumption.

A rigid sampling, e.g., at localities from which exclusively two-spored versus four-spored basidiome populations of *‘C. aegerita* s.l.’ were reported, may yield clarity about the hypothetical presence of monokaryotic fruiters s.str. in nature—an open question for decades of mycological research. Such an approach must, of course, include comparative basidiome morphology assessment and cultivation-based approaches including histology and nuclear state examination. Ultimately, modern -omics, karyotyping, and functional genetics-based approaches may allow us to predict on or reveal the type of reproduction monokaryotic fruiting s.str. constitutes (asexual, unisexual, parasexual?) and in how far it may confer competitive advantages.

## Data Availability

All data generated or analyzed in this study are included in this article and its supplementary information files.

## Supplementary Materials

The following are available online at https://www.mdpi.com/article/10.3390/jof7050394/s1, Figure S1. Visualization of nuclear state and septa allocation in hyphal segments of the monokaryons *C. parasitca* ICMP 11668-1 to -12 (a–l). Mycelia were incubated in micro-cultivation chambers at 25 °C for 4 days, treated with ethanol/acetic acid fixative, stained with DAPI/Calcofluor, and photographed at 1000× magnification applying fluorescence microscopy. Red arrows point to nuclei, barbed blue arrows indicate two septa delimiting a hyphal segment. In some cases (e.g., (c,g,j)), some background staining is detectable. The scale bars represent 10 µm. Figure S2. Fruiting assay with *C. aegerita* AAE-3 ((a), dikaryotic); *C. aegerita* AAE-3-32 ((b), monokaryotic); *C. parasitica* ICMP 11668 ((c), dikaryotic); and ‘mycelium-type’ monokaryons *C. parasitica* ICMP 11668-1, -2, -5, -6, -7, -8, -10, -11, and -12 (d–l) in the fruiting setup of Herzog et al. [18]. Strains were grown on 1.5% MEA for 10-19 days at 25 °C in the dark. Fully colonized plates were fruiting induced at 20 °C in a 12 h light/12 h dark rhythm at saturated humidity in microfilter-aerated wet chambers. The scale bar represents 2 cm. Figure S3. Mating partners of the monokaryotic fruiting structure-forming monokaryons *C. parasitica* ICMP 11668-3, -4, and -9. Compatible mating was established by *C. parasitica* ICMP 11668-3 with *C. parasitica* ICMP 11668-6, -8, and -9. The same outcome applies to mating of *C. parasitica* ICMP 11668-4 with *C. parasitica* ICMP 11668-5. (a) Outgrowth of dikaryotic mycelium from compatible mating reactions on 1.5% MEA plates after 12 days in the dark at 25 °C. The scale represents 2 cm. (b) Clamp connection formation (red arrows) indicates mating compatibility in a respective *C. parasitica* ICMP 11668 progeny sibling mating. The scale represents 10 µm.
