# Supplementary figures and images for: The Pacific Tree-Parasitic Fungus Cyclocybe parasitica Exhibits Monokaryotic Fruiting, Showing Phenotypes Known from Bracket Fungi and from Cyclocybe aegerita
Source: J Fungi (Basel). 2021 May 19;7(5):394. doi: 10.3390/jof7050394 (PMC8159124; doi:10.3390/jof7050394)

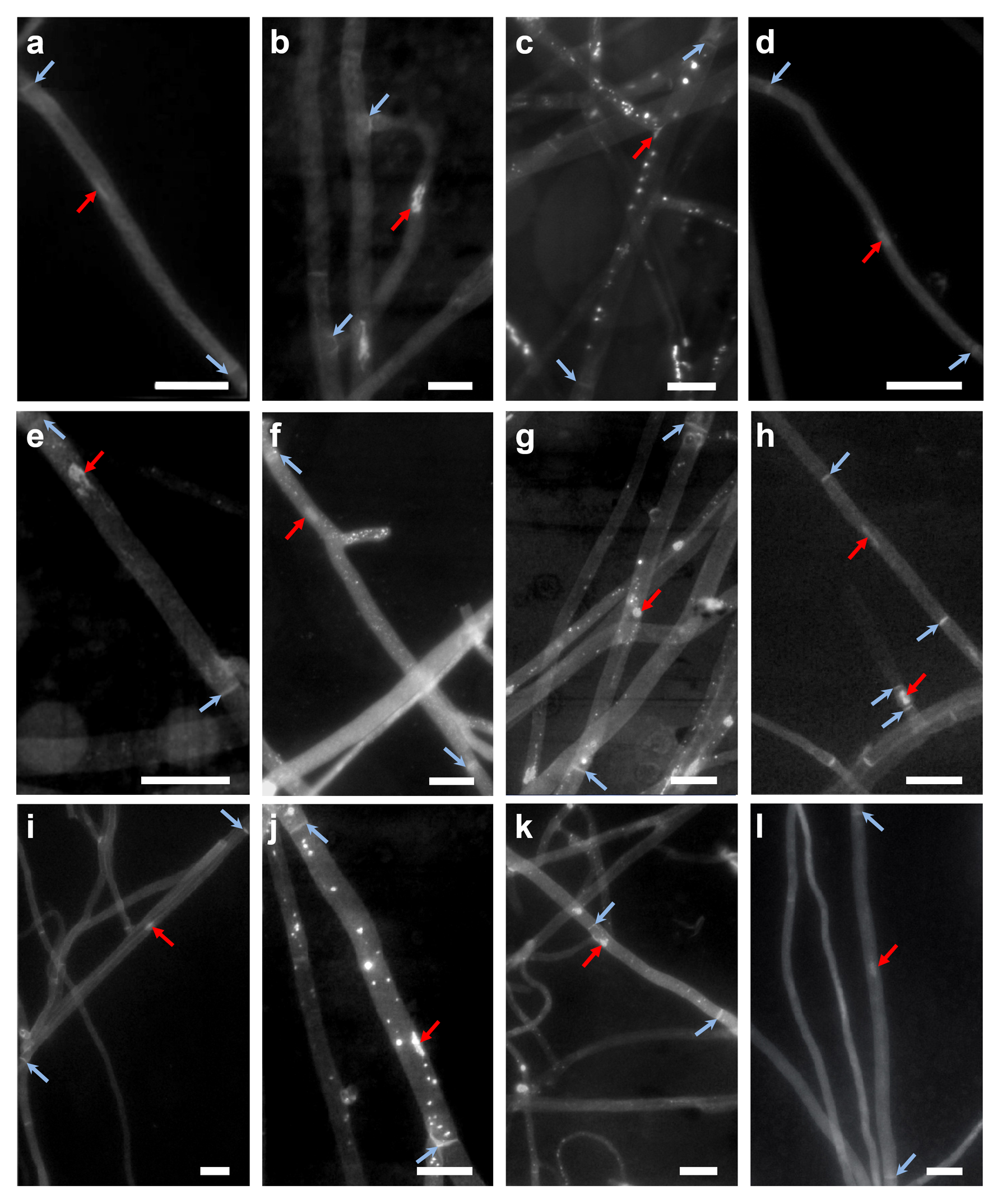

Supplement: Supplementary file 1 [file jof-07-00394-s001.zip › jof-1199556-supplementary_revision/Figure S1_revised.tif]

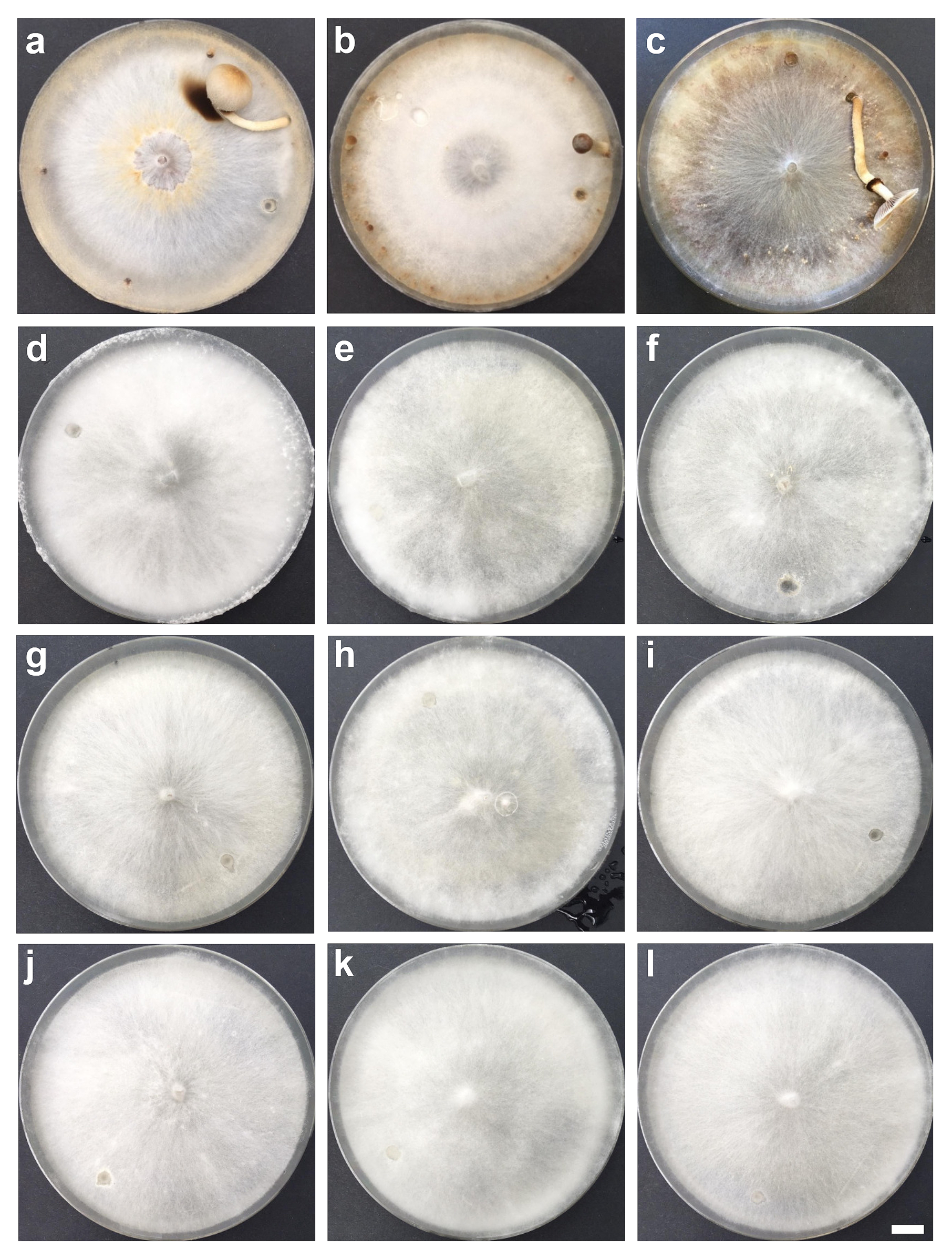

Supplement: Supplementary file 1 [file jof-07-00394-s001.zip › jof-1199556-supplementary_revision/Figure S2.tif]

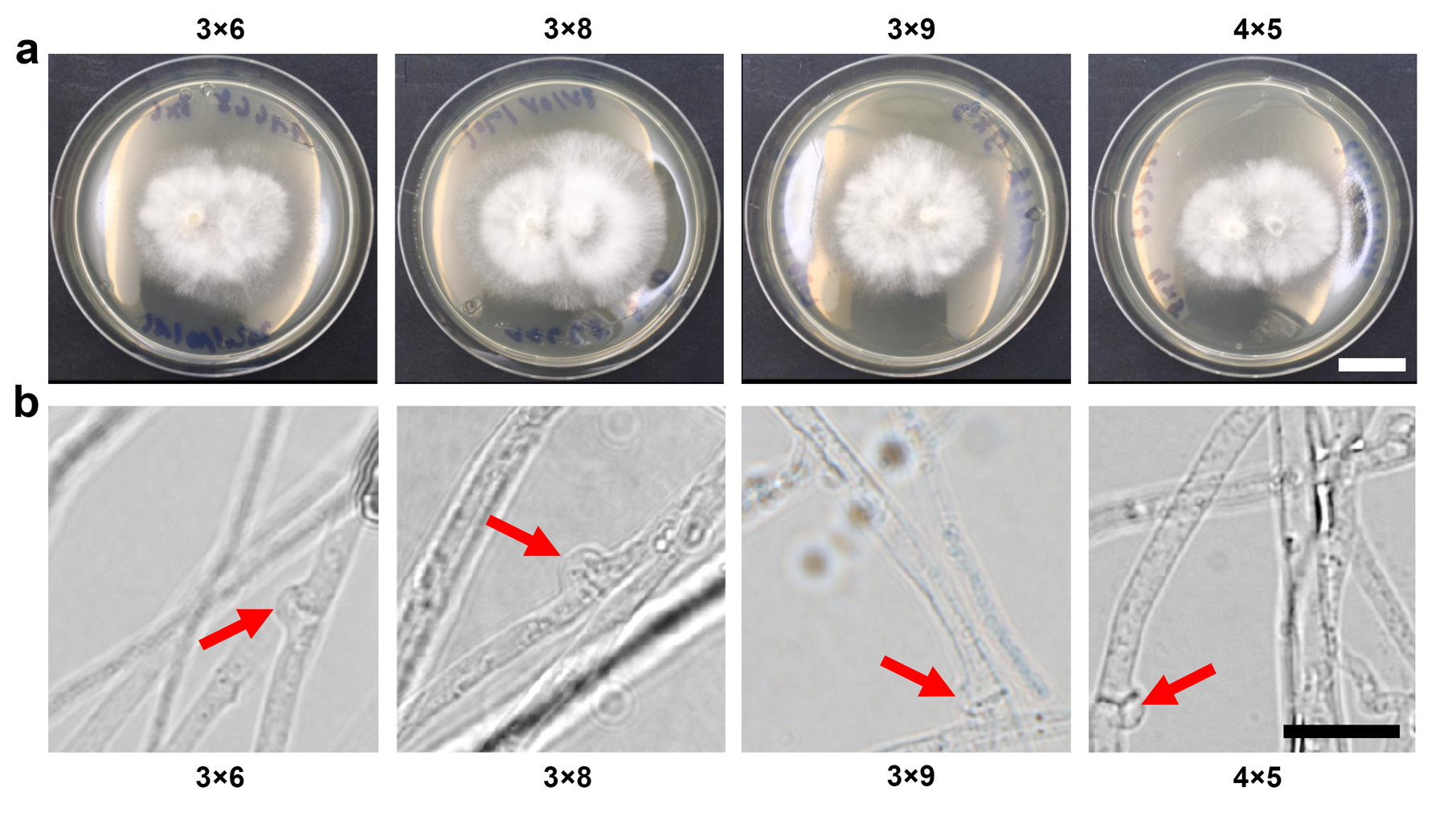

Supplement: Supplementary file 1 [file jof-07-00394-s001.zip › jof-1199556-supplementary_revision/Figure S3.tif]
